# Utilization of Low Molecular Weight Carbon Sources by Fungi and *Saprolegniales*: Implications for Their Ecology and Taxonomy

**DOI:** 10.3390/microorganisms11030782

**Published:** 2023-03-18

**Authors:** Hossein Masigol, Hans-Peter Grossart, Seyedeh Roksana Taheri, Reza Mostowfizadeh-Ghalamfarsa, Mohammad Javad Pourmoghaddam, Ali Chenari Bouket, Seyed Akbar Khodaparast

**Affiliations:** 1 Plankton and Microbial Ecology, Leibniz Institute for Freshwater Ecology and Inland Fisheries (IGB), 16775 Neuglobsow, Germany; hossein.masigol@gmail.com (H.M.); roxi.thi@gmail.com (S.R.T.); 2 Department of Plant Protection, Faculty of Agricultural Sciences, University of Guilan, Rasht 4199613776, Iran; javad.pormoghadam@gmail.com (M.J.P.); blumeria2015@gmail.com (S.A.K.); 3 Institute for Biochemistry and Biology, Potsdam University, 14469 Potsdam, Germany; 4 Department of Plant Protection, School of Agriculture, Shiraz University, Shiraz 7144113131, Iran; rmostofi@shirazu.ac.ir; 5 East Azarbaijan Agricultural and Natural Resources Research and Education Centre, Plant Protection Research Department, Agricultural Research, Education and Extension Organization (AREEO), Tabriz 5355179854, Iran; a.chenari@areeo.ac.ir

**Keywords:** carbon cycling, catabolic potential, eco-physiology, freshwater fungi, freshwater oomycetes, phylogeny, polyphasic taxonomy

## Abstract

Contributions of fungal and oomycete communities to freshwater carbon cycling have received increasing attention in the past years. It has been shown that fungi and oomycetes constitute key players in the organic matter cycling of freshwater ecosystems. Therefore, studying their interactions with dissolved organic matter is crucial for understanding the aquatic carbon cycle. Therefore, we studied the consumption rates of various carbon sources using 17 fungal and 8 oomycete strains recovered from various freshwater ecosystems using EcoPlate™ and FF MicroPlate™ approaches. Furthermore, phylogenetic relationships between strains were determined via single and multigene phylogenetic analyses of the internal transcribed spacer regions. Our results indicated that the studied fungal and oomycete strains could be distinguished based on their carbon utilization patterns, as indicated by their phylogenetic distance. Thereby, some carbon sources had a higher discriminative strength to categorize the studied strains and thus were applied in a polyphasic approach. We concluded that studying the catabolic potential enables a better understanding of taxonomic relationships and ecological roles of fungal vs. oomycete strains.

## 1. Introduction

Freshwater ecosystems are highly dynamic as they constantly interact with their terrestrial surroundings. In particular, they receive substantial loads of organic matter (OM) from the surrounding vegetation, e.g., plant debris [1,2]. As soon as OM enters the water, the process of humification is initiated, in which OM will be exposed to various biochemical processes (by freshwater microbial communities), which results in the degradation as well as transformation of big plant-originated polymers to more recalcitrant compounds such as high molecular weight (HMW) humic substances (HS). Both the quality and quantity of HS are believed to have a stabilizing effect on freshwater ecosystems as they contribute to disease suppression, water and nutrient retention, and growth enhancement [3,4]. The most accepted theory states that the transition from OM to HMW HS usually occurs via two separate pathways. In one path, polymeric-based HMW OM such as lignin, cellulose, hemicellulose, pectin (plant-derived), and chitin (animal-derived) are gradually disintegrated into HS precursors [5]. Unlike bacteria, fungal communities facilitate the production of such precursors due to their exceptional ability to produce a variety of extracellular enzymes and subsequently transform these precursors into HS [6,7]. In the other path, however, low molecular weight (LMW) compounds are leached from OM and are easily utilized by a wide range of microorganisms. Generally, LMW OM accounts for <20% of the entire carbon pool and contributes to biogeochemical processes in two different ways [8]. Firstly, they accelerate the humification process by providing an instant energy source to microbial communities to degrade the more recalcitrant polymers. Secondly, regardless of their role as an available energy source, LWM OM compounds such as sugars and amino acids are part of the humification processes as they constitute intermediate molecules.

Despite the involvement of fungal communities in the degradation of HMW OM macromolecules, knowledge of their interactions with LMW OM compounds is scarce. These interactions are of great ecological importance since the abundance, diversity, and sustainability of fungal communities could be correlated with the available LWM OM compounds in their corresponding ecosystems [9,10]. Therefore, natural or human-made fluctuations in the composition of such compounds can potentially perturb spatiotemporal patterns of fungal communities as well. To better understand the relevance of fungal interactions with LWM OM, more studies are required to determine the significance of LMW OM composition on fungal communities. It is still questionable whether fungal members can be distinguished by their utilization patterns of various LMW OM compounds.

The order *Saprolegniales* (*Oomycota*) represents another eukaryotic group of microorganisms with similar physiological traits to fungi. A large body of evidence suggests that *Saprolegniales* are dominantly associated with various OM compounds in freshwater ecosystems [11,12]. However, due to their destructive impact on some aquatic animals such as fish and crayfish [13,14], most studies focus only on their pathogenicity and ignore other ecological contributions. Our previous investigations suggest that *Saprolegniales* differ from many fungi in lignin degradation, i.e., the inability to produce enzymes related to plant-derived OM processing [15,16]. However, it remains unclear whether *Saprolegniales* strains can be physiologically separated from other oomycete and fungal taxa via their utilization patterns of LMW OM. In fact, one could hypothesize that *Saprolegniales*, similar to other eukaryotic and prokaryotic microorganisms, compete over LMW OM (as a labile source of carbon) by utilizing some compounds more efficiently than others [16]. Therefore, it needs to be determined whether/to what extent LMW OM influences communities of *Saprolegniales* in in a given environment.

The combination of morphometric data and nucleotide sequences with eco-physiological features (known as the polyphasic taxonomic approach) to characterize fungal species has led to a more congruent taxonomic framework [17]. A lack of nucleotide sequences, misassigned sequences, inaccurate/incomplete morphological descriptions, the high variability of morphometric features, and the absence of congruency between DNA sequences and the morphology of many fungal taxa justify why additional eco-physiological traits should be considered when designing a more reliable taxonomy [18,19]. For example, polyphasic taxonomy has been used in large, heterogeneous, and cosmopolitan genera such as *Cladosporium* (Pers.) Link, *Penicillium* Link, and *Aspergillus* P. Micheli to better resolve their taxonomy [20,21,22]. Eco-physiological features in fungal taxa such as the source of isolation, lifestyle, associations with other organisms, and tolerance to environmental parameters have been used to discriminate individual strains of closely related taxa [23,24,25,26]. One of the most promising features in studying fungi is their ability to utilize a variety of organic matter sources with different degrees of bioavailability. Such utilization ability shows whether and/or to what extent specific fungal taxa are involved in carbon and nutrient cycling. Yet, fungal interactions with organic matter remain largely unknown, mainly because a well-established tool for comparative investigations is lacking.

EcoPlate™ is a practical tool used mainly for microbial community analysis. The result of inoculating any given sample in EcoPlate™ plates (with 31 individual carbon sources) will be unique carbon-utilization patterns. These data are used for analyzing (dis)similarities among samples and to identify any probable correlations with their corresponding environments [27,28]. Later, Filamentous Fungi (FF) MicroPlate™, a similar product to EcoPlate™ (with 95 individual carbon sources), was introduced to determine fungal taxonomy from the carbon-utilization pattern of individual strains. However, its application remained limited to mainly community-level physiological profiling [29,30,31]. Therefore, a combined approach is required in which both carbon-utilization potentials and taxonomic affiliation are studied simultaneously.

Recently, EcoPlate™ and FF MicroPlate™ have been used to address restrictions in determining both the function and taxonomy of fungi and oomycetes. In particular, they have been mainly used to examine whether the inter- and intra-species catabolic versatility of fungal and oomycete strains reflect their DNA sequence-based phylogenetic relationships [17]. Determining the metabolic potential of individual fungal strains to utilize specific carbon sources will promote our understanding of the fungal involvement in ecological functions related to nutrient and carbon cycling within the freshwater realm.

We used EcoPlate™ and FF MicroPlate™ tools to investigate the eco-physiological capacity of 17 fungal and 8 oomycete strains isolated from two freshwater ecosystems. In addition, single and multigene phylogenies of the tested strains were constructed to examine whether the strains’ DNA- and eco-physiological-based categorizations match. Our study has important implications for understanding the (dis)similar ecological roles of fungi and oomycetes in freshwater nutrient cycling and their taxonomic variability.

## 2. Materials and Methods

### 2.1. Isolation of Fungal and Saprolegniales Strains

Sampling was conducted in Lake Stechlin, Northeastern Germany, and Anzali lagoon, Northern Iran. Plant debris was collected from the shoreline, transported into the lab, rinsed with sterilized water, cut into equal pieces, and placed in Petri dishes containing a piece of moist cotton towel. The Petri dishes were kept at room temperature and checked for fungal growth. As soon as mycelia or a fungal organ emerged, they were transferred to a potato dextrose agar (PDA) medium (4, 20, and 15 g/L of infused potatoes, dextrose, and agar, respectively). The hyphal tipping method was applied to obtain pure isolates. The cultures were kept at 4 °C for the follow-up experiments [32].

The same materials were used to isolate *Saprolegniales* strains. Approximately equal pieces of plant debris (5 × 5 cm) were placed in Petri dishes containing boiled sterilized hemp seeds as baits. The Petri dishes were monitored daily for any sign of hemp seeds’ colonization by *Saprolegniales* strains. As soon as the mycelia were observed, some small pieces were transported to cornmeal agar (CMA) medium (2 and 15 g/L of infused cornmeal and agar, respectively) using a sterilized needle. The colony grown on the CMA medium (amended with fluconazole and ketoconazole) were sub-cultured at least two times to minimize the risk of fungal and bacterial contaminations. The final cultures were kept at 4 °C for the follow-up experiments [33].

### 2.2. DNA Extraction, PCR and Sequencing

DNA was extracted following the protocol suggested by Montero-Pau et al. [34]. Briefly, sterilized 1.5-mL tubes containing 100 μL of alkaline lysis buffer (NaOH 25 mM/L, disodium ETDA 0.2 mM/L, pH 8.0) were prepared, followed by adding a clot of mycelia and one round of centrifugation for 30 min at 9000 rpm. After a 30-min incubation period at 95 °C, the tubes were cooled on ice for 5 min. At last, 100 μL of neutralizing solution (Tris-HCl 40 mM/L, pH 5.0) was added to each tube and they were stored at −20 °C for the sequencing. Then, ribosomal internal transcribed spacer (ITS) and large subunit (LSU) regions were amplified using the ITS1/ITS4 and LR0R/LR5 primer pairs and the respective PCR conditions [35,36,37] in a thermocycler (Analytikjena, Jena, Germany). The PCR amplification products were then sent to Marcogen company (Amsterdam, The Netherlands) for Sanger sequencing. The accession numbers of sequences were obtained by editing the resulting sequences in BioEdit software [38] and submitting the improved versions to GenBank.

### 2.3. Phylogenetic Analyses

Alignments and phylogenetic analyses of ITS, LSU, and ITS+LSU rDNA sequences using maximum parsimony and maximum likelihood were constructed as described in Masigol et al. [39]. Table S1 summarizes all used strains, their corresponding sequences, and GenBank accession numbers.

### 2.4. Consumption Rates of Various Carbon Sources

Due to higher isolation frequency, all *Cladosporium* spp. and *Penicillium* spp. strains were inoculated in FF MicroPlate™ to make the comparison of their utilization patterns of LMW compounds reliable. However, *Saprolegniales* strains could not be examined using FF MicroPlate™ as their zoospores died after inoculation to wells. The FF MicroPlate™ contains 95 unique carbon source wells and one well containing water as the control. According to Atanasove and Druzhinia [40], carbon sources are categorized into 15 classes: amino acids (12 substrates), glucosides (11), oligosaccharides (10), others (10), polyols (9), hexoses (8), polysaccharides (6), sugar acids (6), TCA-cycle intermediates (5), heterocyclic amines (4), hexosamines (4), pentoses (4), aliphatic organic acids (3), peptides (2), and heptose (1) (Figure S1). This classification was used to determine which classes can better separate *Cladosporium* spp. and *Penicillium* spp. strains.

Spore suspensions were prepared for inoculation in FF MicroPlate™ plates. For fungal strains, 20 mL of FF Inoculating Fluid (Biolog part number 72106) were poured into Petri dishes containing 5–7 days old colonies to facilitate the detachment of propagules. The suspension of propagules was then transferred to falcon tubes for each strain. A hemocytometer was used to create an approximately similar concentration of spores per strain (10^3^ to 10^5^ propagules per 100 μL). Finally, we homogenously transferred 100 μL from the Falcon tubes containing correct concentration of propagules to each well of the plates. The plates were sealed using Parafilm, placed into plastic bags containing moist paper towels, and incubated at 25 °C. The activity in the plates was measured using a microplate reader set for absorbance at 490 nm at 2, 12, 24, 48, 60, 72, 84, 96, and 108 h after inoculation. A reduction in iodonitrophenyltetrazolium redox dye amended in each carbon source well results in the formation of a purple color with maximum absorbance at 490 nm. The measurement was then used to calculate the activity of each strain for each carbon source: difference between the OD of the carbon source containing carbon source wells and the control well at 490 nm.

We used another similar tool named EcoPlate™ for less frequently isolated fungal strains, including species of *Aspergillus*, *Fusarium* (two strains), *Paecilomyces*, *Plectosphaerella*, *Sarocladium*, and *Volutella*. Moreover, EcoPlate™ was used for two *Saprolegniales* isolated genera, namely *Achlya* (three strains) and *Dictyuchus* (five strains), as they could produce viable zoospores in wells. Additionally, 31 carbon sources were classified into five categories: amines/amides, amino acids, carbohydrates, carboxylic and ketonic acid, and polymers (Figure S2).

The inoculation of fungal and *Saprolegniales* strains into EcoPlate™ plates was similar to the process explained above (Section 2.4), with some differences as follows: For *Saprolegniales* strains, the method by Unestam [41] was used to yield spore suspension. Briefly, a small piece of agar from the pure cultures was transferred to new Petri dishes containing 5 mL of liquid PG1 medium. After four days of incubation at 18 °C, 2 mL of autoclaved natural water was used to wash hyphal biomass grown on the medium three times. The hyphal biomass was then transferred to another Petri dish containing 4 mL of autoclaved natural water for 24 h at 18 °C. As soon as zoospores emerged in the liquid and the target concentration was reached, they were transferred to wells of plates. The activity in the plates was measured using the microplate reader set for absorbance at 590 nm at 0, 24, 48, 72, 96, 120, 168, 192, and 216 h after inoculation. Reducing the iodonitrophenyltetrazolium redox dye amended in each carbon source well results in the formation of a purple color with maximum absorbance at 590 nm. The measurements were the same as explained above.

### 2.5. Statistical Analysis

The activity of fungal and *Saprolegniales* strains in each carbon source well at different time points was measured using a microplate reader to determine the difference between the optical density of the carbon-source-containing wells and the control well as explained above. Each measurement was repeated three times for EcoPlate™ (in the same 95 well plate) and twice for FF MicroPlate™ (in two separate 95 well plates). Moreover, SPSS 16.0 software was used to run linear discriminant analysis to determine whether/how carbon sources (separately and together) (95 and 31 variables in EcoPlate™ and FF MicroPlate™, respectively) can separate fungal and *Saprolegniales* strains (5 and 15 classes in EcoPlate™ and FF MicroPlate™, respectively) in accordance with their phylogenetic relationships. The analysis resulted in two linear dimensions (LD1 and LD2), which illustrate how many percentages of variance in the activity of strains on each/a group of carbon sources can be explained by their phylogenetic relationships. The last five measurements (average from the replicates) were incorporated in all analyses (activity at 60, 72, 84, 96, and 108 h after inoculation for FF MicroPlate™ and 120, 144, 168, 192, and 216 h after inoculation for EcoPlate™). The same data set was also used for constructing the dendrogram based upon 31 and 95 carbon sources in EcoPlate™ and FF MicroPlate™, respectively.

## 3. Results

### 3.1. Taxonomy and Phylogeny of Fungal and Saprolegniales Strains

**Figure 1:**
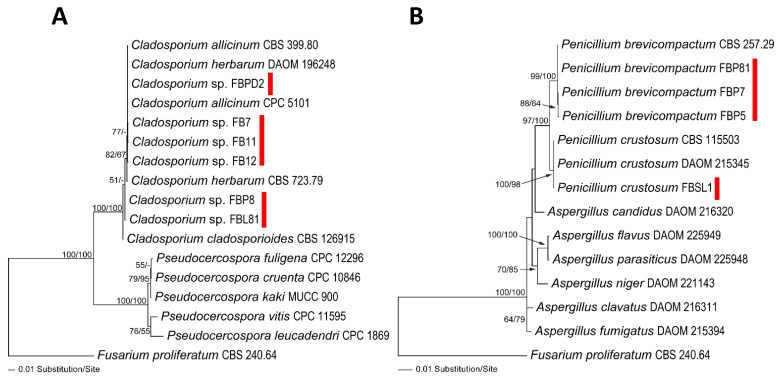
Phylogram of the best ML trees revealed by RAxML from an analysis of the combined ITS–LSU for *Cladosporium* (**A**) and *Penicillium* (**B**) strains isolated in this study (lnL = −3527.0288 and −4195.2300, respectively). *Fusarium proliferatum* (CBS 240.64) was considered the outgroup. ML and MP bootstrap supports above 50% were given at the first and second positions, respectively, above or below the branches. Red marks are strains *Cladosporium* and *Penicillium* isolated in this study.

**Figure 2:**
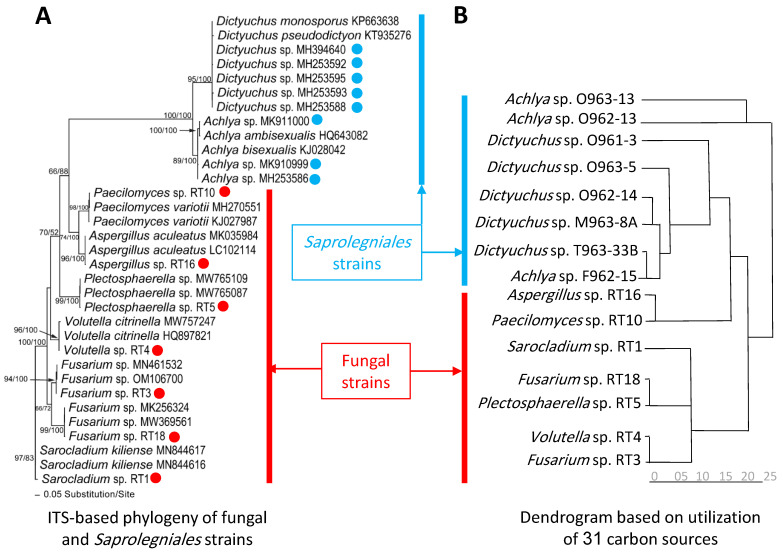
Phylogram of the best ML trees (lnL = −4501.7999) revealed using RAxML from an analysis of ITS (**A**) and the dendrogram (**B**) based on utilization of 31 carbon sources of seven fungal and eight oomycete strains and related taxa. The analyses for constructing the dendrogram were performed based on the last five measurements (average from the replicates). Blue and red circles represent *Saprolegniales* and fungal strains isolated in this study. ML and MP bootstrap supports above 50% were given at the first and second positions, respectively, above or below the branches.

The amplified regions of ITS and LSU were used to determine the phylogenetic position of fungal and *Saprolegniales* strains (only ITS) (Figure 1A,B and Figure 2A). Accordingly, *Cladosporium* spp. strains FBPD2, FB11, FB12, and FB7 were associated with *Cladosporium herbarum* (Pers.) Link and *Cladosporium allicinum* (Fr.) Bensch, U. Braun and Crous in the *C. herbarum* complex while *Cladosporium* sp. FBL81 and FBP8 were more closely related to *Cladosporium cladosporioides* (Fresen.) G.A. de Vries in the C. *cladosporioides* complex. Additionally, *Penicillium* spp. strains FBP5, FBP7, and FBP81 had the closest similarity to *Penicillium brevicompactum* Dierckx and strain FBSL1 to *Penicillium crustosum* Thom. Moreover, the strains *Sarocladium* sp. RT1, *Fusarium* sp. (RT3 and RT18), *Volutella* sp. RT4, *Plectosphaerella* sp. RT5, *Paecilomyces* sp. RT10, and *Aspergillus* sp. RT16 were phylogenetically associated with *Sarocladium kiliense* (Grütz) Summerb., an unknown *Fusarium* taxa, *Volutella citronella* (Cooke and Massee) Seifert, unknown *Plectosphaerella* taxa, *Paecilomyces variotii* Bainier, and *Aspergillus aculeatus* Iizuka, respectively (Figure 2A). *Saprolegniales* strains were associated with *Achlya* (three strains) and *Dictyuchus* taxa (five strains) (Figure 2A).

Additionally, Figure 2 showed that the constructed dendrogram based on the utilization rate of 31 carbon sources in EcoPlate™ could well separate most strains phylogenetically assigned to fungi and *Saprolegniales* (Table S1, measurements related to the mean difference between the OD of the carbon source containing wells and the control well for the last five time points (60, 72, 84, 96, and 108 h after inoculation)). In particular, all *Saprolegniales* were grouped with the exception of two *Achlya* spp. strains, creating a separate group. Similarly, five fungal strains were placed in one group, except for *Aspergillus* sp. RT16 and *Peacilomyces* sp. RT10, which showed carbon utilization capabilities more similar to *Saprolegniales*.

### 3.2. Carbon Utilization Using Cladosporium *spp*. and Penicillium *spp*. Strains in FF MicroPlate™

**Figure 3:**
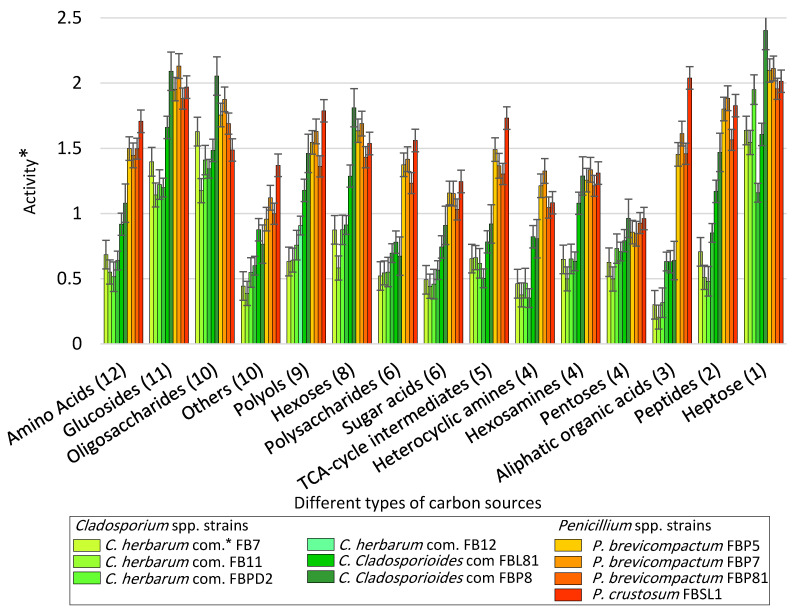
The average utilization rate of 15 classes of carbon sources after 108 h using *Cladosporium* spp. and *Penicillium* spp. strains inoculated to the commercial FF MicroPlate™ plates (See Figure S1 for more details about carbon sources) based upon the categorization style of Atanasove and Druzhinia [40] (*com. = complex, error bars = standard error) (* = Optical density (OD) of carbon source wells inoculated with strains—OD of the control well at 590 nm).

Figure 3 provides a comparison of the average utilization rate of 95 substrates (categorized in 15 classes) using the tested *Cladosporium* spp. and *Penicillium* spp. Strains (Table S2; measurements related to the mean difference between the OD of the carbon source-containing wells and the control well for the last five time points at 490 nm (120, 144, 168, 192, and 216 h after inoculation). In general, glucosides, oligosaccharides, and heptose were the most favorable carbon source categories, as their utilization rate, by all strains of *Cladosporium* and *Penicillium* species, were above average. In contrast, the utilization rate of pentoses and heterocyclic amines were below average by all strains (except for *Penicillium brevicompactum* FBP5 and FBP7). *Penicillium* spp. strains showed higher utilization rates than *Cladosporium* spp. concerning seven categories, including amino acids, polysaccharides, sugar acids, TCA-cycle intermediates, heterocyclic amines, aliphatic organic acids, and peptides. *Cladosporium* spp. and *Penicillium* spp. strains differed the most from each other by their utilization rate of Aliphatic organic acids.

*Cladosporium cladosporioides* FBP8 and *P. crustosum* FBSL1 were the most active strains: while the level of utilization in *C. cladosporioides* FBP8 was the highest with respect to four categories (oligosaccharides, hexoses, pentoses, and heptose), six other categories (amino acids, polyols, polysaccharides, sugar acids, TCA-cycle intermediates, and aliphatic organic acids) were utilized most by *P. crustosum* FBSL1. Moreover, *C. herbarum* FB11 and *P. brevicompactum* FBP81 were the least active strains: while the utilization rate in *C. herbarum* FB11 was the lowest with respect to three categories (sugar acids, heterocyclic amines, and aliphatic organic acids), pentoses were utilized the least by *P. brevicompactum* FBP81.

Additionally, the degree of the metabolic overlaps, with respect to the utilization rate of 95 substrates, was studied. Figure S3 reveals strong differences in both intra- and inter-genus levels. The average utilization rate of strains FBPD2, FB11, FB12, and FB7 (phylogenetically placed in the C. *herbarum* complex) were compared against FBL81 and FBP8 (phylogenetically placed in the C. *cladosporioides* complex) (Figure S3A). The same comparison was conducted between strains FBP5, FBP7, and FBP81 (phylogenetically identified as *P. brevicompactum*) and FBSL1 (phylogenetically identified as *P. crustosum*) (Figure S3B). Finally, all *Cladosporium* spp. Strains were compared against the *Penicillium* spp. Strains. Both *Cladosporium* spp. And *Penicillium* spp. Strains showed diverse average utilization rates for 15 classes of substrates; moreover, they showed a range of utilization rates with respect to the substrates in each class (S3C).

### 3.3. Carbon Utilization Using Fungal vs. Saprolegniales Strains in EcoPlate™

**Figure 4:**
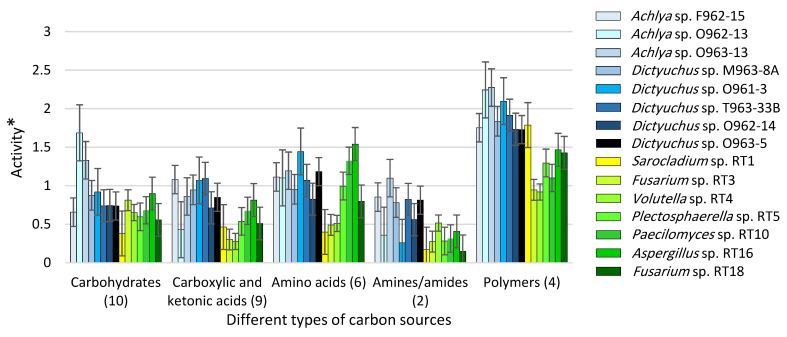
The activity of fungal and *Saprolegniales* strains inoculated to the EcoPlate™ plates after 216 h (see Figure S2 for more details about the specific carbon sources) (* = Optical density (OD) of carbon source wells inoculated with strains—OD of the control well at 590 nm).

Figure 4 illustrates the utilization level of five categories of carbon sources using seven fungal and eight *Saprolegniales* strains. The levels of utilization of polymers and amines/amides were the highest and lowest regarding both fungal and *Saprolegniales* strains, respectively. In general, *Saprolegniales* were more active than fungal strains considering all carbon source categories. Among *Saprolegniales*, *Achlya* sp. O963-13 and *Dictyuchus* sp. O962-14 were the most and least active strains, respectively. Moreover, *Aspergillus* RT16 and *Fusarium* RT3 showed the highest and lowest levels of substrate utilization among all fungal strains, respectively. Moreover, the degree of the metabolic overlaps, with respect to the utilization rate of 31 substrates, was studied. The differences between *Achlya* spp. and *Dictyuchus* spp. strains as well as fungal and *Saprolegniales* strains, are presented in Figure S4A,B.

### 3.4. Discriminative Potential of Carbon Source Categories in FF MicroPlate™

**Figure 5:**
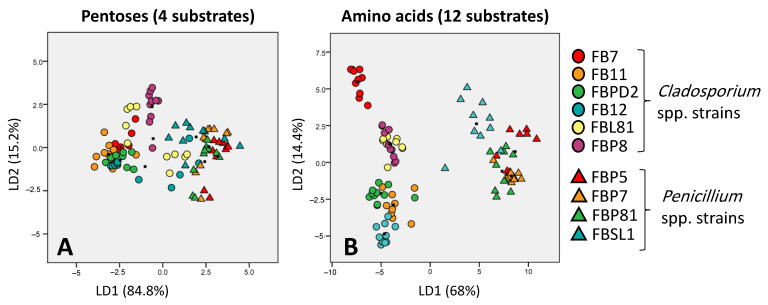
Discriminative ability of pentoses (the worst discriminator) (**A**) and amino acids (the best discriminator) (**B**) in separating six and four *Cladosporium* spp. and *Penicillium* spp. strains, respectively; based on Atanasove and Druzhinia [40] categorization. LD1 and LD2 show that many percentages of variance observed in the dataset are explained using linear dimension 1 and linear dimension 2.

Based on Atanasove and Druzhinia [40] categorization, amino acids, oligosaccharides, and polysaccharides were the most promising categories in discriminating fungal strains according to their carbon utilization pattern. In contrast, peptides, pentoses, and sugar acids categories showed the weakest potential to discriminate strains correctly (Figure 5).

### 3.5. Discrimination Potential of Carbon Source Categories in EcoPlate™

**Figure 6:**
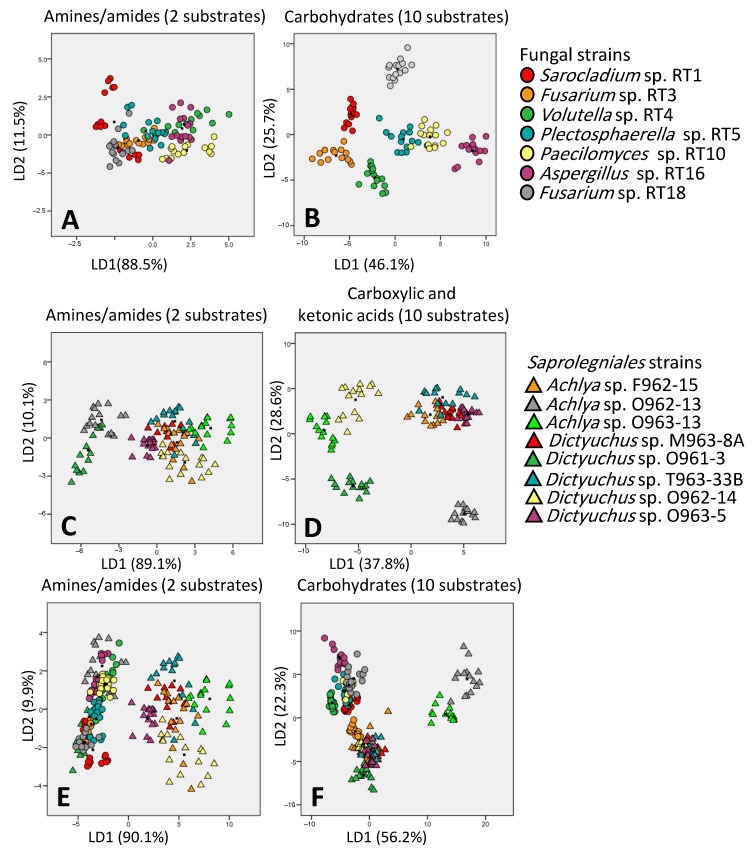
Discrimination ability of amides/amines ((**A**) for fungal, (**C**,**E**) for *Saprolegniales* strains) and carbohydrates ((**B**) for fungal, (**D**,**F**) for *Saprolegniales* strains) asthe worst and best discriminators, respectively. LD1 and LD2 show how many percentages of variance observed in the dataset are explained using linear dimension 1 and linear dimension 2.

The discrimination ability of carbon source categories was investigated firstly for fungal strains, then for *Saprolegniales* strains, and finally for both together. For fungal and *Saprolegniales* strains, the categories of carbohydrates and carboxylic and ketonic acids showed the best discrimination ability, respectively. When analyzed together, fungal and Saprolegniales strains were separated the best and worst by amid/amines and carbohydrates, respectively (Figure 6).

**Figure 7:**
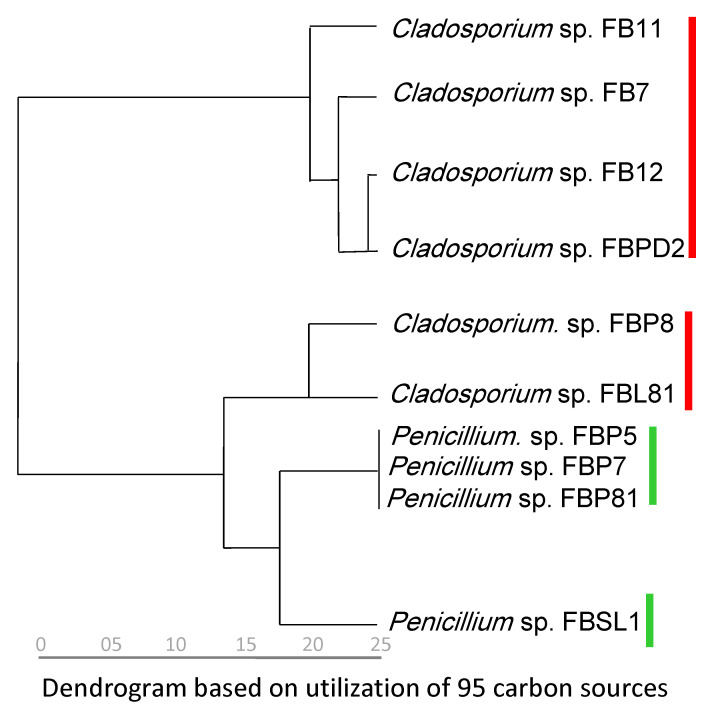
Dendrogram inferred from the utilization of 95 carbon sources. The analyses for constructing the dendrogram were performed based on the last five measurements (average from the replicates). Red and green marks are *Cladosporium* and *Penicillium* strains isolated in this study, respectively.

Categorization of strains based on phylogenetic relationship (the combined tree) and carbon utilization capabilities (95 sources in FF MicroPlate™) resulted in a similar grouping. *Cladosporium* spp. strains were divided into two groups according to their carbon utilization capabilities, similar to the phylogenetic trees which separated *Cladosporium* spp. strains into two clades. Moreover, the phylogenetic separation of *Penicillium* sp. FBSL1 (assigned to *P. crustosum*) from three others (FBP5, FBP81, and FBP7 assigned to *P. brevicompactum*) was confirmed by the dendrogram that divided *Penicillium* spp. strains similarly (Figure 7).

## 4. Discussion

This study investigated the contribution of fungal and *Saprolegniales* strains in the utilization of carbon sources using two recently developed tools, EcoPlate™ and FF MicroPlate™. Accordingly, we drew two important conclusions: Firstly, we showed that fungi and *Saprolegniales* strains prefer some LMW carbon sources over others, even though most of these sources are easily accessible in freshwater ecosystems. Secondly, we illustrated that EcoPlate™ and FF MicroPlate™ could be complementary tools for systematic and phylogenetic studies of fungi and *Saprolegniales*.

The humification of dissolved organic matter occurs in three stages: (I) initial decomposition of labile carbon sources, (II) slow decomposition, and (III) direct genesis and degradation of more recalcitrant compounds [42]. While only fungi and a few other microorganisms are involved in the latter two stages [15,16,43], diverse communities (both prokaryotes and eukaryotes) use labile carbon as easily accessible sources of energy. In our study, fungal strains utilized labile sources differently in terms of the utilization rate and diversity of consumed OM sources, which might suggest a niche partitioning through source availability. Similarly, Hanson et al. [44] showed that fungal communities are changed in response to various labile carbon sources (such as glycine and sucrose) and may specialize in breaking down particular OM compounds. In other words, LMW carbon sources from various OM sources might alter fungal communities.

LMW OM includes both natural sources (such as LMW drainage from natural landscapes and its production by microbial biomass) [45] and anthropogenic sources (e.g., outflows from urbanized and intensively farmed agricultural landscapes) [46]. Some of these compounds might be toxic for some fungi but favorable for others. Therefore, fluctuations in LMW carbon sources potentially cause micro-niches, in which some fungal communities dominate the others. Chigineva et al. [47] demonstrated that, after adding sucrose as a labile carbon source, the relative abundance of *Cladosporium* and *Penicillium* taxa increased and decreased, respectively. Such an alteration in the abundance and diversity of fungal communities influenced by labile carbon sources has also been shown by Ren et al. [48]. Therefore, it can be argued that, similar to the previously observed impact of dissolved OM on fungal distribution and diversity [49,50], the composition of labile carbon sources also changes the functional structure and competitive ability of saprotrophic fungal communities.

Additionally, based upon a rather limited number of strains in this study, we could confirm that both Ecoplate™ and FF MicroPlate™ tools are practical in the taxonomy of fungal strains. Some labile carbon sources, such as amino acids and carbohydrates, are useful in separating strains within and between taxa. Moreover, the dendrograms of fungal strains based upon the utilization rate of all 31 and 95 carbon sources were generally in accordance with the constructed phylogenetic trees. In contrast to our study, where phylogeny and ecology based categorizations of *Cladosporium* spp. and *Penicillium* spp. strains greatly matched, Kubicek et al. [51] and Barrera et al. [52] showed that carbon utilization patterns of 10 *Cladorrhinum* spp. and 21 *Trichoderma* spp. strains did not correspond to the taxonomic delimitation of the species, respectively. Therefore, an approach which contains both phylogeny and ecology must be tested on larger sets of fungal strains for a better taxonomic resolution. Moreover, it must be clarified that fungal strains isolated in this study do not fall into the classical definition of “freshwater fungi” as most of them have a cosmopolitan nature. Therefore, it is important to address, firstly, whether they are active players within the boundaries of freshwater ecosystems and, secondly, how abundant they are. These two aspects will determine how scholars might deal with cosmopolitan fungal species in freshwater ecosystems.

Oomycetes were considered a fungal group for more than a century due to many similarities in morphology and lifestyle. These similarities, however, were the result of convergent evolution and not their evolutionary relatedness to each other. Oomycetes and fungi have distinct cellular traits and evolutionary history as they are currently placed in distant lineages in the tree of life [53]. Here, we suggested that, despite the co-occurrence of *Saprolegniales* with fungi in various freshwater habitats [54], they tend to minimize competition over labile carbon sources. This was proven by separating most of our *Saprolegniales* from fungal strains based on their carbon utilization patterns, which also reflects their distant phylogenetic relationship. Therefore, the contrasting role of oomycetes and fungi in the degradation of large recalcitrant polymers such as lignin [15] should be extended to labile organic matter. Nevertheless, more studies are needed, particularly in other unstudied Iranian freshwater ecosystems [55], as our results were limited to only two *Saprolegniales* genera, *Achlya* and *Dictyuchus*.

## 5. Conclusions

Fungal and oomycetes constitute a major fraction of heterotrophic microbial communities in freshwater ecosystems. These communities are key carbon and nutrient cycling regulators due to their close association with organic matter and energy flow in freshwater food webs. We showed that, although fungi and oomycetes have both colonized freshwater ecosystems for millions of years, they behave differently with respect to the utilization of labile carbon sources. Such a different behavior might originate from their distant phylogenic relationship. Our findings have both ecological and taxonomic implications which need to be addressed in the future: (I) It seems that fungal and oomycete communities tend to minimize their competition for labile organic matter by utilizing various types of carbon sources and, as a result, receive enough energy to proceed with their contribution in the decomposition of more recalcitrant organic matter. This is how these communities serve their ecosystem by fueling entire food webs and carrying energy to all trophic levels. Assuming the distinct utilization of labile carbon sources by fungi vs. oomycetes, one could argue that fluctuations in the environmental parameters of freshwater ecosystems (i.e., origin, type, and quantity of labile carbon source) might eventually cause a shift in the spatio-temporal distribution of fungal and oomycete communities. (II) In our study, we observed intra-taxa variability in both fungal and oomycete strains for their interaction with labile organic matter. In particular, *Cladosporium* and *Penicillium* strains from fungi and *Achlya* and *Dictyuchus* strains from oomycetes showed intra-taxa variability even though they were phylogenetically similar. This highlights the use of eco-physiological traits of fungi and oomycetes to overcome the inconsistencies regarding their taxonomy. Thus, a robust tool is required to enable comparative studies. We propose that EcoPlate™ and FF MicroPlate™ are able to reflect eco-physiological differences between various strains, especially ecologically diverse cosmopolitan genera, and can be established as valuable eco-taxonomic tools for further studies.

## Data Availability

The datasets used and/or analyzed during the current study are available from the corresponding authors on request. Moreover, sequences were deposited in GenBank (http://www.ncbi.nlm.nih.gov/, accessed on 9 January 2023) under the accession numbers mentioned in the text.

## Supplementary Materials

The following supporting information can be downloaded at https://www.mdpi.com/article/10.3390/microorganisms11030782/s1, Table S1: Isolation and accession numbers of sequences used in the phylogenetic analyses; Table S2: Measurements related to the mean difference between the OD at 590 nm of the carbon source containing wells and the control well for the last five time points (120, 144, 168, 192, and 216 h after) inoculation); Table S3: Measurements related to the mean difference between the OD at 590 nm of the carbon source containing wells and the control well for the last five time points (60, 72, 84, 96, and 108 h after inoculation); Figure S1: Categorization of 95 carbon sources FF MicroPlate™ based on Atanasova and Druzhinina, 2010; Figure S2: Categorization of 31 carbon sources in EcoPlate™. Figure S3: Scatter plots of activity for *Cladosporium* spp. strains (A), *Penicillium* spp. strains (B), and both genera (C) (* = Optical density (OD) of carbon source wells inoculated with strains—OD of the control well at 490 nm); Figure S4: Scatter plots of activity for *Achlya* vs. *Dictyuchus* strains (A) and *Saprolegniales* vs. fungal strains (B) (* = Optical density (OD) of carbon source wells inoculated with strains—OD of the control well at 490 nm). References [56,57,58,59,60,61,62,63,64,65,66,67] are cited in the supplementary materials.
