# Supplementary material for: Utilization of Low Molecular Weight Carbon Sources by Fungi and Saprolegniales: Implications for Their Ecology and Taxonomy
Source: Microorganisms. 2023 Mar 18;11(3):782. doi: 10.3390/microorganisms11030782 (PMC10052706; doi:10.3390/microorganisms11030782)
Supplement: Supplementary file 1 [file microorganisms-11-00782-s001.zip › Figure S1 to S4.pptx]

## Slide 1
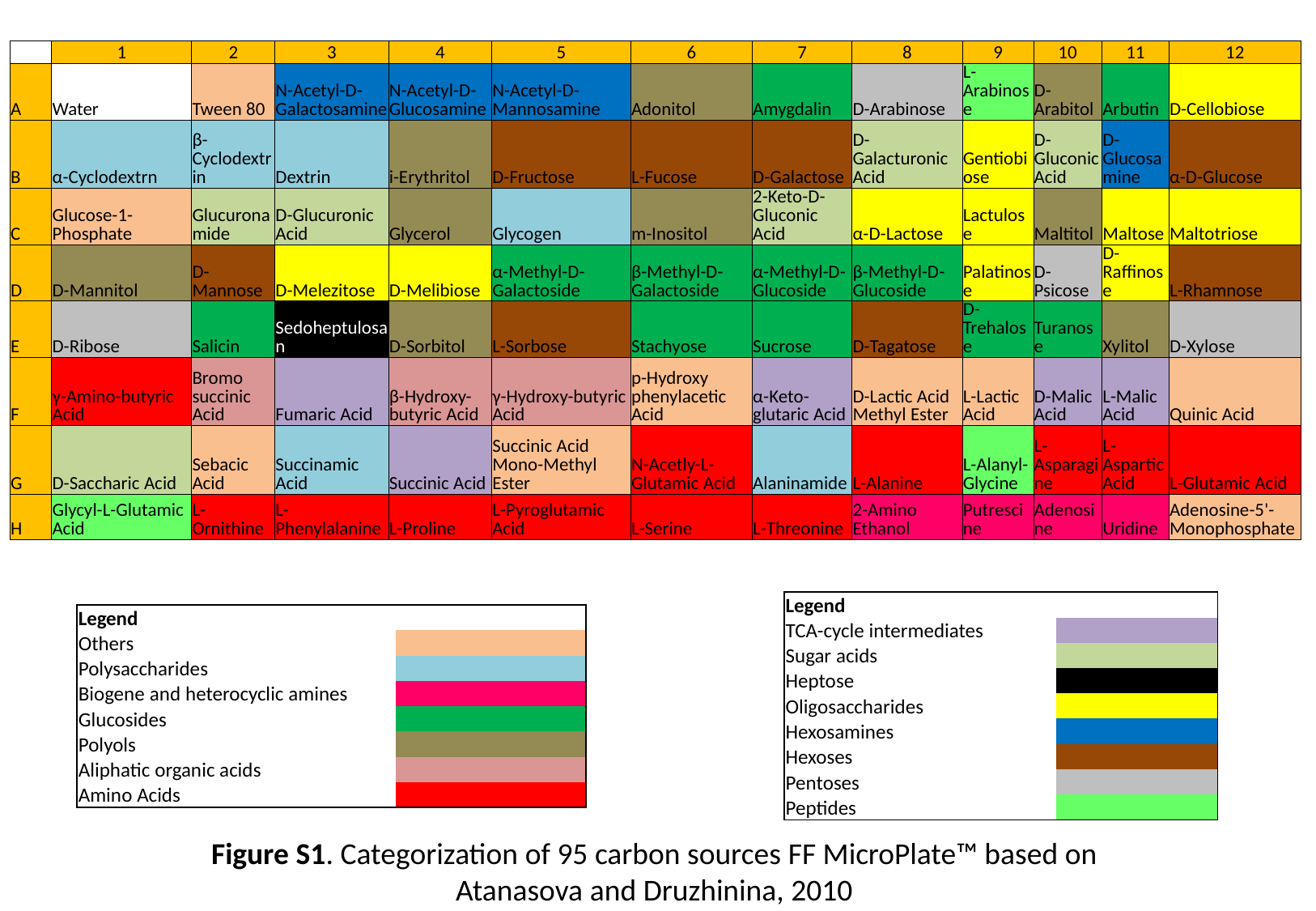

| | 1 | 2 | 3 | 4 | 5 | 6 | 7 | 8 | 9 | 10 | 11 | 12 |
| --- | --- | --- | --- | --- | --- | --- | --- | --- | --- | --- | --- | --- |
| A | Water | Tween 80 | N-Acetyl-D-Galactosamine | N-Acetyl-D-Glucosamine | N-Acetyl-D-Mannosamine | Adonitol | Amygdalin | D-Arabinose | L-Arabinose | D-Arabitol | Arbutin | D-Cellobiose |
| B | α-Cyclodextrn | β-Cyclodextrin | Dextrin | i-Erythritol | D-Fructose | L-Fucose | D-Galactose | D-Galacturonic Acid | Gentiobiose | D-Gluconic Acid | D-Glucosamine | α-D-Glucose |
| C | Glucose-1-Phosphate | Glucuronamide | D-Glucuronic Acid | Glycerol | Glycogen | m-Inositol | 2-Keto-D-Gluconic Acid | α-D-Lactose | Lactulose | Maltitol | Maltose | Maltotriose |
| D | D-Mannitol | D-Mannose | D-Melezitose | D-Melibiose | α-Methyl-D-Galactoside | β-Methyl-D-Galactoside | α-Methyl-D-Glucoside | β-Methyl-D-Glucoside | Palatinose | D-Psicose | D-Raffinose | L-Rhamnose |
| E | D-Ribose | Salicin | Sedoheptulosan | D-Sorbitol | L-Sorbose | Stachyose | Sucrose | D-Tagatose | D-Trehalose | Turanose | Xylitol | D-Xylose |
| F | γ-Amino-butyric Acid | Bromo succinic Acid | Fumaric Acid | β-Hydroxy-butyric Acid | γ-Hydroxy-butyric Acid | p-Hydroxy phenylacetic Acid | α-Keto-glutaric Acid | D-Lactic Acid Methyl Ester | L-Lactic Acid | D-Malic Acid | L-Malic Acid | Quinic Acid |
| G | D-Saccharic Acid | Sebacic Acid | Succinamic Acid | Succinic Acid | Succinic Acid Mono-Methyl Ester | N-Acetly-L-Glutamic Acid | Alaninamide | L-Alanine | L-Alanyl-Glycine | L-Asparagine | L-Aspartic Acid | L-Glutamic Acid |
| H | Glycyl-L-Glutamic Acid | L-Ornithine | L-Phenylalanine | L-Proline | L-Pyroglutamic Acid | L-Serine | L-Threonine | 2-Amino Ethanol | Putrescine | Adenosine | Uridine | Adenosine-5'-Monophosphate |
| Legend | |
| --- | --- |
| TCA-cycle intermediates | |
| Sugar acids | |
| Heptose | |
| Oligosaccharides | |
| Hexosamines | |
| Hexoses | |
| Pentoses | |
| Peptides | |
| Legend | |
| --- | --- |
| Others | |
| Polysaccharides | |
| Biogene and heterocyclic amines | |
| Glucosides | |
| Polyols | |
| Aliphatic organic acids | |
| Amino Acids | |
Figure S1. Categorization of 95 carbon sources FF MicroPlate™ based on
Atanasova and Druzhinina, 2010

## Slide 2
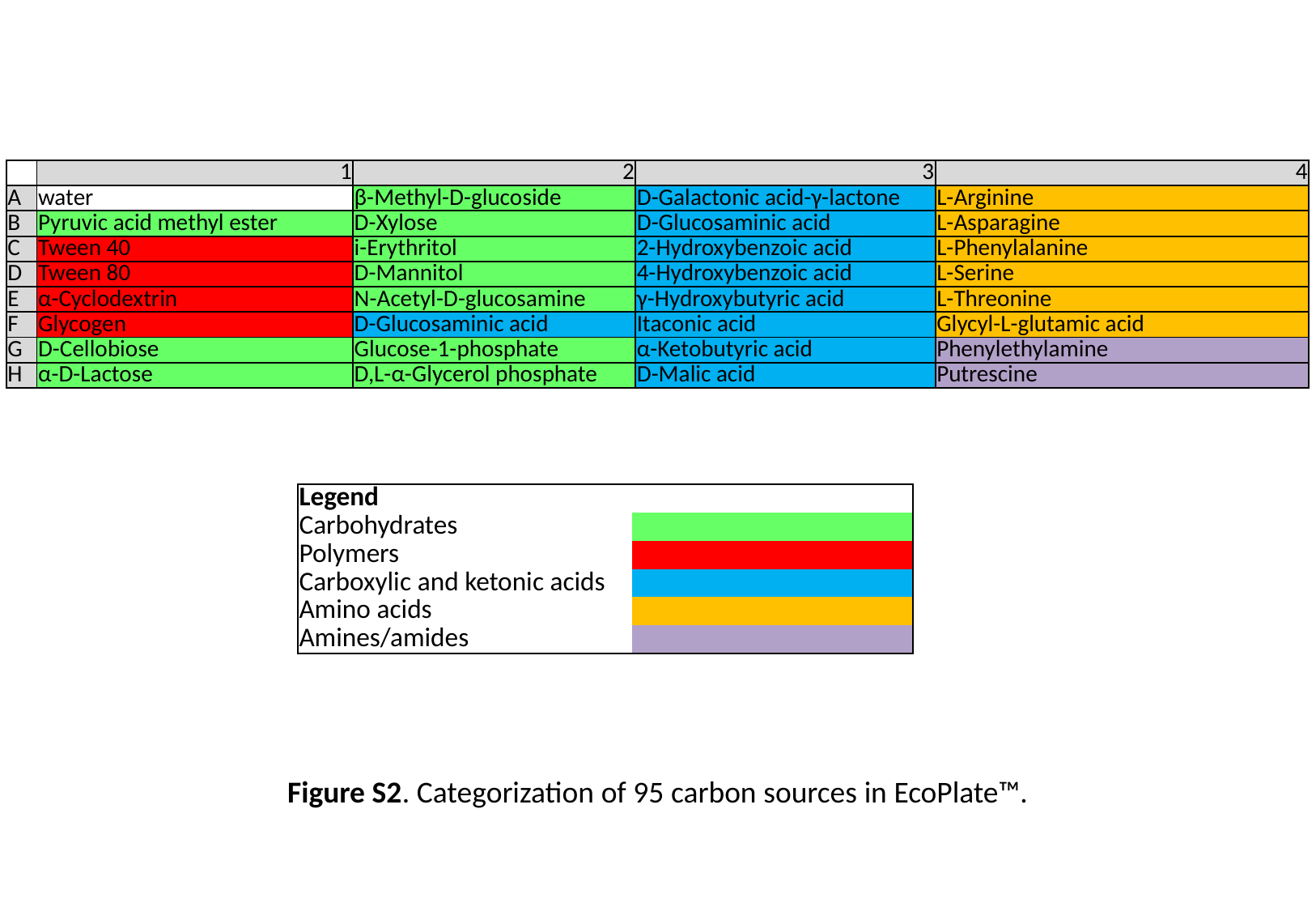

| | 1 | 2 | 3 | 4 |
| --- | --- | --- | --- | --- |
| A | water | β-Methyl-D-glucoside | D-Galactonic acid-γ-lactone | L-Arginine |
| B | Pyruvic acid methyl ester | D-Xylose | D-Glucosaminic acid | L-Asparagine |
| C | Tween 40 | i-Erythritol | 2-Hydroxybenzoic acid | L-Phenylalanine |
| D | Tween 80 | D-Mannitol | 4-Hydroxybenzoic acid | L-Serine |
| E | α-Cyclodextrin | N-Acetyl-D-glucosamine | γ-Hydroxybutyric acid | L-Threonine |
| F | Glycogen | D-Glucosaminic acid | Itaconic acid | Glycyl-L-glutamic acid |
| G | D-Cellobiose | Glucose-1-phosphate | α-Ketobutyric acid | Phenylethylamine |
| H | α-D-Lactose | D,L-α-Glycerol phosphate | D-Malic acid | Putrescine |
| Legend | |
| --- | --- |
| Carbohydrates | |
| Polymers | |
| Carboxylic and ketonic acids | |
| Amino acids | |
| Amines/amides | |
Figure S2. Categorization of 95 carbon sources in EcoPlate™.

## Slide 3
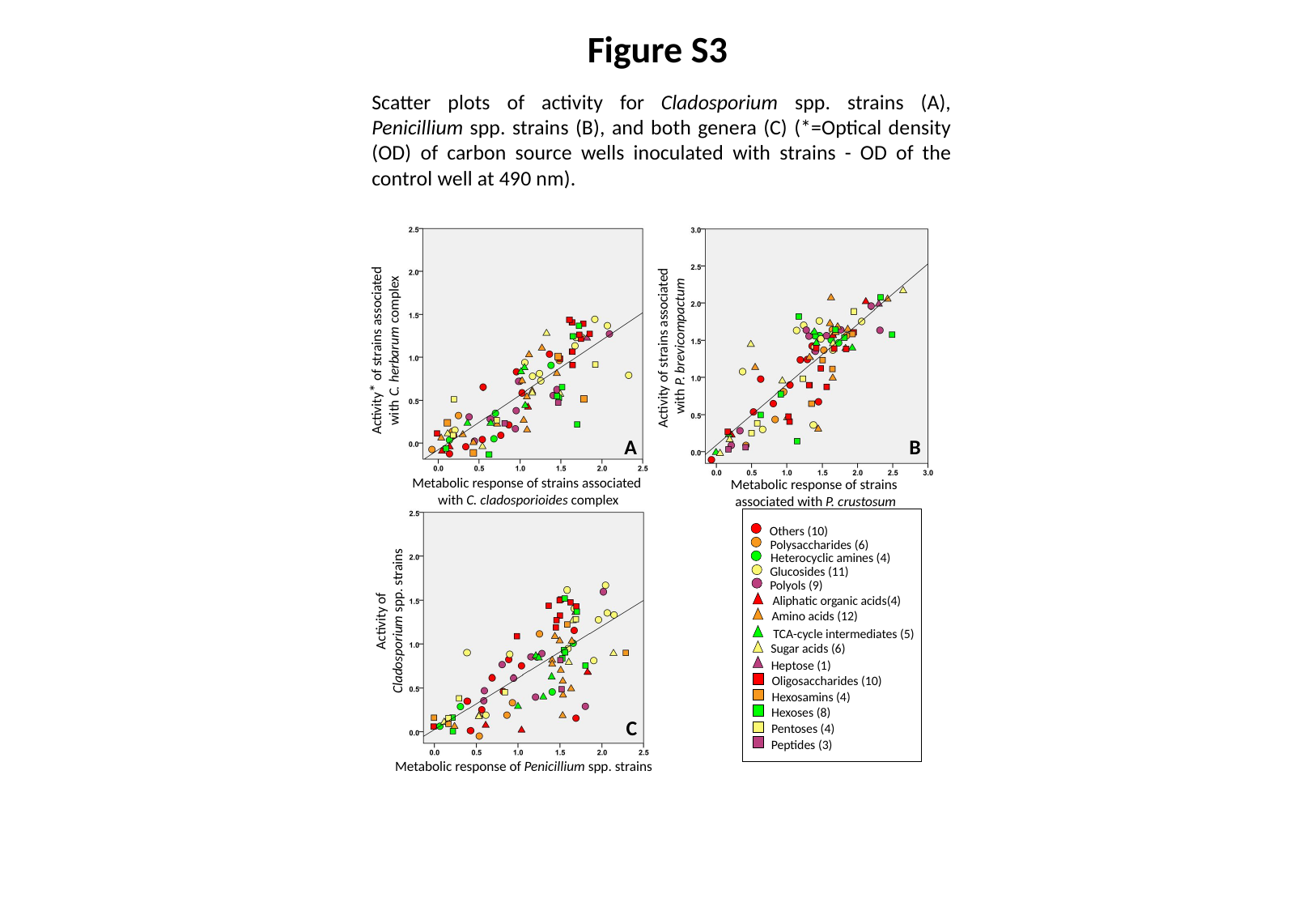

Figure S3
Scatter plots of activity for Cladosporium spp. strains (A), Penicillium spp. strains (B), and both genera (C) (*=Optical density (OD) of carbon source wells inoculated with strains - OD of the control well at 490 nm).
Activity of strains associated
with P. brevicompactum
Activity* of strains associated
with C. herbarum complex
B
A
Metabolic response of strains associated
with C. cladosporioides complex
Metabolic response of strains
associated with P. crustosum
Others (10)
Polysaccharides (6)
Heterocyclic amines (4)
Glucosides (11)
Polyols (9)
Aliphatic organic acids(4)
Activity of
Cladosporium spp. strains
Amino acids (12)
TCA-cycle intermediates (5)
Sugar acids (6)
Heptose (1)
Oligosaccharides (10)
Hexosamins (4)
Hexoses (8)
C
Pentoses (4)
Peptides (3)
Metabolic response of Penicillium spp. strains

## Slide 4
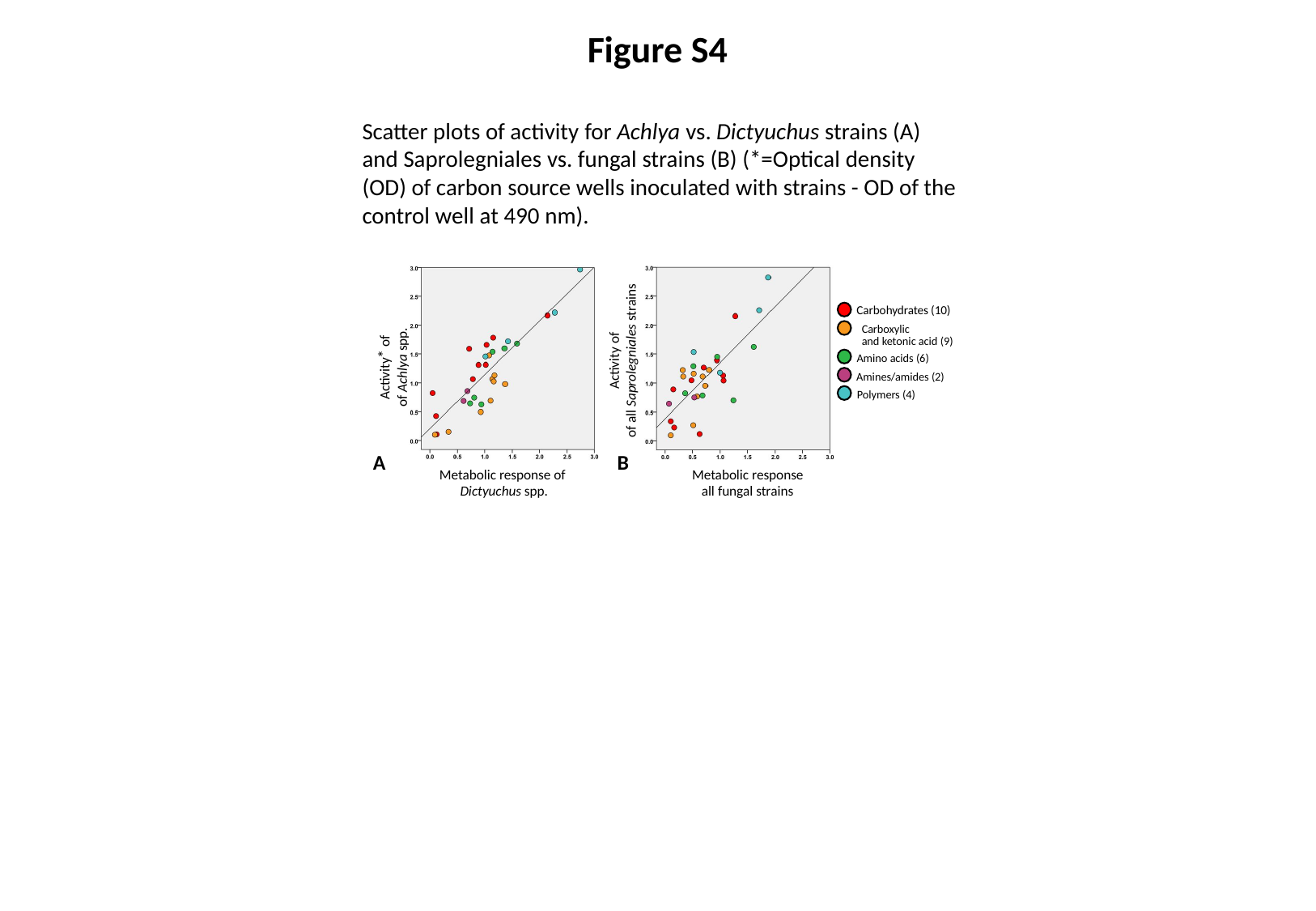

Figure S4
Scatter plots of activity for Achlya vs. Dictyuchus strains (A) and Saprolegniales vs. fungal strains (B) (*=Optical density (OD) of carbon source wells inoculated with strains - OD of the control well at 490 nm).
Carbohydrates (10)
Carboxylic
and ketonic acid (9)
Activity of
of all Saprolegniales strains
Activity* of
of Achlya spp.
Amino acids (6)
Amines/amides (2)
Polymers (4)
A
B
Metabolic response of
Dictyuchus spp.
Metabolic response all fungal strains
