# Supplementary material for: Utilization of Low Molecular Weight Carbon Sources by Fungi and Saprolegniales: Implications for Their Ecology and Taxonomy
Source: Microorganisms. 2023 Mar 18;11(3):782. doi: 10.3390/microorganisms11030782 (PMC10052706; doi:10.3390/microorganisms11030782)
Supplement: Supplementary file 1 [file microorganisms-11-00782-s001.zip › Table S1_new.pdf]

Supporting Information

Utilization of Low Molecular Weight Carbon Sources by Fungi and *Saprolegniales*: Implications for Their Ecology and Taxonomy

Hossein Masigol [1,2], Hans-Peter Grossart [1,3,*], Seyedeh Roksana Taheri [1], Reza Mostowfizadeh-Ghalamfarsa [4], Mohammad Javad Pourmoghaddam [2], Ali Chenari Bouket [5] and Seyed Akbar Khodaparast [2]

[1] Plankton and Microbial Ecology, Leibniz Institute for Freshwater Ecology and Inland Fisheries (IGB), 16775 Neuglobsow, Germany; hossein.masigol@gmail.com (H.M.); roxi.thi@gmail.com (S.R.T.)
[2] Department of Plant Protection, Faculty of Agricultural Sciences, University of Guilan, Rasht 4199613776, Iran; javad.pormoghadam@gmail.com (M.J.P.); blumeria2015@gmail.com (S.A.K.)
[3] Institute for Biochemistry and Biology, Potsdam University, 14469 Potsdam, Germany
[4] Department of Plant Protection, School of Agriculture, Shiraz University, Shiraz 7144113131, Iran; rmostofi@shirazu.ac.ir
[5] East Azarbaijan Agricultural and Natural Resources Research and Education Centre, Plant Protection Research Department, Agricultural Research, Education and Extension Organization (AREEO), Tabriz 5355179854, Iran; a.chenari@areeo.ac.ir
* Correspondence: hgrossart@igb-berlin.de; Tel.: +49-(0)-3308269991

LIST OF SUPPORTING INFORMATION

Table S1. Isolation and accession numbers of sequences used in the phylogenetic analyses ……................................…. 3
References to Table S1………………………………………………..……………………..………………..……. 5

**Table S1.** Isolation and accession numbers of sequences used in the phylogenetic analyses. Isolates/sequences in bold were isolated/sequenced in present study. N/A: not available.				
**Species**	**Strain number**	**GenBank accession number**		**References**
		**ITS**	**LSU**	
Achlya ambisexualis	CBS38379	HQ643082	N/A	Robideau et al. (2011)
Achlya bisexualis	GHL2012.2	**KJ028042**	N/A	unpublished
Achlya sp.	**F962-15**	**MH394644**	N/A	**This study**
Achlya sp.	**O963-13**	**MH253591**	N/A	**This study**
Achlya sp.	**O962-13**	**MH253586**	N/A	**This study**
Aspergillus aculatus	F1P3RSF4	MK035984	N/A	unpublished
Aspergillus aculatus	KKU-CT2	LC102114	N/A	unpublished
Aspergillus candidus	DAOM 216320	JN942868	JN938922	Schoch et al. (2012)
Aspergillus clavatus	DAOM 216311	JN942917	JN938924	Schoch et al. (2012)
Aspergillus flavus	DAOM 225949	JN942867	JN938926	Schoch et al. (2012)
Aspergillus fumigatus	DAOM 215394	JN942916	JN938928	Schoch et al. (2012)
Aspergillus niger	DAOM 221143	JN942866	JN938930	Schoch et al. (2012)
Aspergillus parasiticus	DAOM 225948	JN942865	JN938934	Schoch et al. (2012)
Aspergillus sp.	**RT16**	**MH367066**	N/A	**This study**
Cladosporium allicinum	CBS 399.80	AJ244227	DQ678074	Schoch et al. (2006), de Hoog et al. (1999)
Cladosporium allicinum	CPC 5101	AY251078	GU214408	Braun et al. (2003); Crous et al. (2009)
Cladosporium cladosporioides	CBS 126915	MH864338	MH875782	Vu et al. (2019)
Cladosporium cladosporioides	**FBP8**	**MN401025**	**ON211939**	**This study**
Cladosporium cladosporioides	**FBL81**	**MN401020**	**MN396219**	**This study**
Cladosporium herbarum	DAOM 196248	JN942903	JN938886	Schoch et al. (2012)
Cladosporium herbarum	CBS 723.79	EU167558	GU214410	Simon and Weiß (2008); Simon et al. (2009); Crous et al. (2009)
Dictyuchus monosporus	278B_AH	KP663638	N/A	unpublished
Dictyuchus pseudodiktyon	CCIBt 4108	KT935276	N/A	unpublished
Dictyuchus sp.	**M963-8A**	**MH253582**	N/A	**This study**
Dictyuchus sp.	**O962-14**	**MH253592**	N/A	**This study**
Dictyuchus sp.	**O963-5**	**MH253588**	N/A	**This study**
Dictyuchus sp.	**O961-3**	**MH394640**	N/A	**This study**
Dictyuchus sp.	**T963-33B**	**MH253593**	N/A	**This study**
Fusarium proliferatum	CBS 240.64	MH858428	MH870056	Vu et al. (2019)
Fusarium sp.	MRR4	MN461532	N/A	unpublished
Fusarium sp.	brs1	OM106700	N/A	unpublished
Fusarium sp.	-	MK256324	N/A	unpublished
Fusarium sp.	-	MW369561	N/A	unpublished

Table S1. Cont.

**Species**	**Strain number**	**GenBank accession number**		**References**
		**ITS**	**LSU**	
***Fusarium* sp.**	**RT3**	**MH367054**	N/A	**This study**
***Fusarium* sp.**	**RT18**	**MH367068**	N/A	**This study**
Paecilomyces varioti	NR23	MH270551	N/A	unpublished
Paecilomyces varioti	SICAU SDT12	KJ027987	N/A	unpublished
Paecilomyces sp.	**RT10**	**MH367061**	N/A	**This study**
Penicillium brevicompactum	**FBP81**	**MN401026**	**MN396223**	**This study**
Penicillium brevicompactum	**FBP7**	**MN401023**	**MN396222**	**This study**
Penicillium brevicompactum	**FBP5**	**MN401017**	**MN396215**	**This study**
Penicillium crustosum	CBS 115503	MH862985	NG069876	Vu et al. (2019)
Penicillium crustosum	DAOM 215345	JN942857	JN938953	Schoch et al. (2012)
Penicillium crustosum	**FBSL1**	**MN401024**	**ON211940**	**This study**
Plectosphaerella sp.	TV26-T-1	MW765109	N/A	unpublished
Plectosphaerella sp.	TV14-10-1	MW765087	N/A	unpublished
***Plectosphaerella* sp.**	**RT5**	**MH367056**	N/A	**This study**
Pseudocercospora cruenta	CPC 10846	GU269688	GU214673	Crous et al. (2013)
Pseudocercospora fuligena	CPC 12296	GU269711	GU214675	Crous et al. (2013)
Pseudocercospora kaki	MUCC 900	GU269729	GU253761	Crous et al. (2013)
Pseudocercospora leucadendri	CPC 1869	GU269842	GU214480	Crous et al. (2013)
Pseudocercospora vitis	CPC 11595	GU269829	GU214483	Crous et al. (2013)
Sarocladium kiliense	KoLRI_EL005555	MN844617	N/A	unpublished
Sarocladium kiliense	KoLRI_EL005551	MN844616	N/A	unpublished
***Sarocladium* sp.**	**RT1**	**MH367052**	N/A	**This study**
Volutella citrinella	-	HQ897821	N/A	Grafenhan et al. (2011)
Volutella citrinella	CNUFC DYR1	MW757247	N/A	unpublished
***Volutella* sp.**	**RT4**	**MH367055**	N/A	**This study**

References to Table S1

Braun, U.; Crous, P.W.; Dugan, F.; Groenewald, J.Z.; de Hoog, G.S. Phylogeny and taxonomy of *Cladosporium*-like hyphomycetes, including *Davidiella* gen. nov., the teleomorph of *Cladosporium s. str. Mycol. Progress.* **2003**, *2*, 3–18. https://doi.org/10.1007/s11557-006-0039-2

Crous, P.W.; Schoch, C.L.; Hyde, K.D.; Wood, A.R.; Gueidan, C.; de Hoog, G.S.; Groenewald, J.Z. Phylogenetic lineages in the Capnodiales. *Stud. Mycol.* **2009**, *64*, 17–47. https://doi.org/10.3114/sim.2009.64.02

Crous, P.W.; Braun, U.; Hunter, G.C.; Wingfield, M.J.; Verkley, G.J.M.; Shin, H.D.; Nakashima, C.; Groenewald, J.Z. Phylogenetic lineages in *Pseudocercospora*. *Stud. Mycol.* **2013**, *75*, 37–114. http://dx.doi.org/10.3114/sim0005

de Hoog, G.S.; Zalar, P.; Urzì, C.; de Leo, F.; Yurlova, N.A.; Sterflinger, K. Relationships of dothideaceous black yeasts and meristematic fungi based on 5.8S and ITS2 rDNA sequence comparison. *Stud. Mycol.* **1999**, *43*, 31–37.

Gräfenhan, T.; Schroers, H.-J.; Nirenberg, H.I.; Seifert, K.A. An overview of the taxonomy, phylogeny, and typification of nectriaceous fungi in *Cosmospora*, *Acremonium*, *Fusarium*, *Stilbella*, and *Volutella*. *Stud. Mycol.* **2011**, *68*, 79–113. https://doi.org/10.3114/sim.2011.68.04

Lecellier, A.; Mounier, J.; Gaydou, V.; Castrec, L.; Barbier, G.; Ablain, W.; Manfait, M.; Toubas, D.; Sockalingum, G.D. Differentiation and identification of filamentous fungi by high-throughput FTIR spectroscopic analysis of mycelia. *Int. J. Food. Microbiol.* **2014**, *168–169*, 32–41. https://doi.org/10.1016/j.ijfoodmicro.2013.10.011.

Robideau, G.P.; De Cock, A.W.; Coffey, M.D.; Voglmayr, H.; Brouwer, H.; Bala, K.; Chitty, D.W.; Desaulniers, N.; Eggertson, Q.A.; Gachon, C.M.; HU, C.H. DNA barcoding of oomycetes with cytochrome c oxidase subunit I and internal transcribed spacer. *Molecular ecology resources* **2011**, *11(6)*, 1002–1011. https://doi.org/10.1111/j.1755-0998.2011.03041.x

Schoch, C.L.; Shoemaker, R.A.; Seifert, K.A.; Hambleton, S.; Spatafora, J.W.; Crous, P.W. A multigene phylogeny of the Dothideomycetes using four nuclear loci. *Mycologia* **2006**, *98*, 1041–1052. https://doi.org/10.1080/15572536.2006.11832632

Schoch, C.L.; Seifert, K.A.; Huhndorf, S. et al. Nuclear ribosomal internal transcribed spacer (ITS) region as a universal DNA barcode marker for Fungi. *PNAS* **2012**, *109*, 1–6. https://doi.org/10.1073/pnas.1117018109

Simon, U.K.; Weiß, M. Intragenomic Variation of Fungal Ribosomal Genes Is Higher than Previously Thought, *Molecular Biology and Evolution* **2008**, *25(11)*, 2251–2254. https://doi.org/10.1093/molbev/msn188

Simon, U.K.; Groenewald, J.Z.; Crous, P.W. *Cymadothea trifolii*, an obligate biotrophic leaf parasite of *Trifolium*, belongs to Mycosphaerellaceae as shown by nuclear ribosomal DNA analyses. *Persoonia* **2009**, *22*, 49–55. doi:10.3767/003158509X425350

Vu, D.; Groenewald, M.; de Vries, M.; Gehrmann, T.; Stielow, B. et al. Large-scale generation and analysis of filamentous fungal DNA barcodes boosts coverage for kingdom fungi and reveals thresholds for fungal species and higher taxon delimitation. *Stud. Mycol.* **2019**, *92*, 135–154. https://doi.org/10.1016/j.simyco.2018.05.001
